# A Study on the Competencies and Characteristics Required by Intergenerational Programming Professionals

**DOI:** 10.1093/geroni/igaf122.3148

**Published:** 2025-12-31

**Authors:** Stephanie Yu-Ching Chen

**Affiliations:** National Chung Cheng University, Minxiong, Chiayi, Taiwan (Republic of China)

## Abstract

Taiwan faces the challenge of a low birth rate and an aging population, resulting in social problems such as labor shortages and generational conflicts. How to promote cross-generational communication and solidarity has received attention. Intergenerational programs are gradually being promoted in various fields with the support of different government departments. However, there is still a lack of consensus on the professionalism and quality of such projects. This study used face-to-face interviews with 25 scholars and practitioners with experience in intergenerational programming to understand the characteristics and professional abilities required of intergenerational programming professionals. The research results found that intergenerational programming professionals mainly include three roles: leaders, managers, and lecturers. Their main task is to promote interaction, learning, and solidarity across generations. To competently promote intergenerational programs, the promoter should have certain characteristics, including being warm and enthusiastic, easy to interact with others, open and inclusive, flexible and adaptable, and innovative and flexible. Due to the differences in their roles, they need to have the general and the professional capabilities for intergenerational programs. Including professional knowledge such as aging literacy, life course and development, intergenerational learning and intergenerational program; having the attitude of caring and investing in intergenerational issues, respecting multiculturalism, liking both the old adults and children, valuing fairness between generations, and liking teamwork; and having the skills of resource connection and integration, intergenerational communication, promoting intergenerational interaction and connection, and intergenerational program planning and evaluation. These traits and abilities are the key to the success of intergenerational programs.

